# Epigenetic Mechanisms Regulate Stem Cell Expressed Genes *Pou5f1* and *Gfra1* in a Male Germ Cell Line

**DOI:** 10.1371/journal.pone.0012727

**Published:** 2010-09-14

**Authors:** Maren Godmann, Erin May, Sarah Kimmins

**Affiliations:** 1 Department of Animal Science, McGill University, Ste-Anne-de-Bellevue, Quebec, Canada; 2 Department of Pharmacology and Therapeutics, McGill University, Montreal, Quebec, Canada; Ludwig-Maximilians-Universität München, Germany

## Abstract

Male fertility is declining and an underlying cause may be due to environment-epigenetic interactions in developing sperm, yet nothing is known of how the epigenome controls gene expression in sperm development. Histone methylation and acetylation are dynamically regulated in spermatogenesis and are sensitive to the environment. Our objectives were to determine how histone H3 methylation and acetylation contribute to the regulation of key genes in spermatogenesis. A germ cell line, GC-1, was exposed to either the control, or the chromatin modifying drugs tranylcypromine (T), an inhibitor of the histone H3 demethylase KDM1 (lysine specific demethylase 1), or trichostatin (TSA), an inhibitor of histone deacetylases, (HDAC). Quantitative PCR (qPCR) was used to identify genes that were sensitive to treatment. As a control for specificity the *Myod1* (myogenic differentiation 1) gene was analyzed. Chromatin immunoprecipitation (ChIP) followed by qPCR was used to measure histone H3 methylation and acetylation at the promoters of target genes and the control, *Myod1*. Remarkably, the chromatin modifying treatment specifically induced the expression of spermatogonia expressed genes *Pou5f1* and *Gfra1*. ChIP-qPCR revealed that induction of gene expression was associated with a gain in gene activating histone H3 methylation and acetylation in *Pou5f1* and *Gfra1* promoters, whereas CpG DNA methylation was not affected. Our data implicate a critical role for histone H3 methylation and acetylation in the regulation of genes expressed by spermatogonia – here, predominantly mediated by HDAC-containing protein complexes.

## Introduction

The formation of spermatozoa from spermatogonial stem cells throughout adult life is dependent on a tightly orchestrated cell differentiation process governed by unique transcriptional programs and extensive chromatin remodeling [1]. While current information indicates that male germ cell differentiation is driven by tight transcriptional regulation [2], [3], [4], [5], [6], the epigenetic modifications and chromatin remodelers underlying the control of this gene expression are unknown. The epigenetic layer includes modification of histones such as methylation, acetylation, phosphorylation among others, and DNA methylation [7]. Recent large scale genome profiling experiments have revealed general roles for histone modifications in gene regulation whereby histone H3 methylation on lysine 4 (K4) is gene activating, and methylation on lysine 9 (K9) is gene silencing [8], [9]. Overall histone acetylation is associated to an open chromatin state and active gene transcription [10], [11], [12], [13], [14]. Advancements on the readout of these specific marks during cell differentiation are just now being made. For example as embryonic stem cells (ESC) progress from a pluripotent state to one of specialization, this process is characterized by the reconfiguration of developmental genes from a bivalent marking consisting of activating H3K4 trimethylation and repressive H3K27 trimethylation, to a loss of repressive marks and a gain in gene function [15]. Furthermore, there is evidence that epigenetic mechanisms such as DNA methylation underlie the control of expression of key genes such as *Pou5f1* (POU domain class 5 transcription factor 1, also known as *Oct4*) and *Nanog* [16], [17].

In the testis, the production of haploid male gametes includes three phases: mitotic proliferation of spermatogonia, meiotic reshuffling of the genome to create genetic diversity and spermiogenesis, where unique morphological changes occur to transform a haploid, immotile round spermatid to an elongated spermatozoa, capable of fertilizing an oocyte [18], [19]. Spermatogonial stem cells either self-renew or differentiate and the lifetime production of mature spermatozoa is dependent on these stem cells [20], [21]. Inadequate establishment or depletion of the stem cell pool will lead to reduced sperm output and infertility [2], [3], [5]. Knockout and knockdown studies have revealed several transcription factors and proteins involved in signal transduction that are key regulators of stem cell biology including *Pou5f1* and glial cell line derived neurotrophic factor family receptor alpha 1 (*Gfra1*, also known as GDNF-family receptor α1) [4], [22], [23], [24]. The *Pou5f1* transcription factor is strongly expressed in spermatogonia and its ablation results in apoptosis of primordial germ cells. As spermatogonia differentiate *Pou5f1* expression is downregulated [6], [25], [26]. GDNF-family receptor α1 localizes to type A spermatogonia including A single, A paired and A aligned and it is co-expressed with *Pou5f1* [4]. Interestingly, knockdown of *Gfra1* in the testicular stem cell population induced a phenotypic switch towards spermatogonial differentiation as indicated by a decrease in proliferation and expression of *Pou5f1*, as well as an increase in differentiation markers. Moreover GDNF cell-signaling is critical in spermatogonial stem cell renewal [4], [27]. The epigenetic mechanisms involved in regulating these key genes in spermatogenesis are unknown.

Accumulating evidence from gene knockouts has shown that chromatin modifiers such as histone demethylases, histone methyltransferases and histone deacetylases have a critical role in male germ cell development [28], [29], [30], [31], [32], [33], [34]. However information on gene-specific targets and how these chromatin modifiers regulate transcriptional processes and differentiation in spermatogenesis is lacking.

We and others have previously determined that in developing male germ cells histone methylation is highly dynamic and tightly controlled [35], [36]. It is well established that the histone H3 demethylase KDM1 (also known as LSD1/AOF2/BHC110) and the histone deacetylase 1 (HDAC1) form a repressor complex and that their histone modification activities are intimately linked [37], [38], [39]. Recently, we have shown that KDM1 is highly expressed in spermatogonia and acts in a repressor complex containing HDAC1 [35]. KDM1 is known to remove activating mono- and dimethylation marks from H3K4, which results in repression of gene transcription [40]. Conversely, in concert with the androgen receptor KDM1 targets repressive H3K9 mono- and dimethylation marks thereby activating target genes [41].

Our working hypothesis was that by inhibiting chromatin modifiers, KDM1 and HDAC, we would alter the epigenetic landscape of a cultured male germ cell line, GC-1 [42], and in turn change gene expression to reveal genes that are regulated by histone methylation and acetylation. Our specific aim was to identify key genes in spermatogenesis that may be targets of this complex and in turn regulated by histone methylation and acetylation. From a panel of genes known to be expressed in spermatogenesis we showed that the spermatogonia expressed genes *Pou5f1* and *Gfra1* were sensitive to the chromatin modifying treatment. We then examined how the expression of these genes was influenced by the levels of histone H3 methylation and acetylation, and DNA methylation in the gene promoters.

## Materials and Methods

### Cell Culture

The GC-1 cell line was obtained from ATCC (CRL-2053; Manassas, VA, USA). GC-1 cells were generated by Hofmann et al. and originally derived from postnatal day 10 mouse testes. They have been described as an intermediate spermatogenic cell type between type B spermatogonia and preleptotene spermatocytes due to morphological and gene expression characteristics [42]. The cells were cultured in DMEM (11965; Invitrogen, Carlsbad, CA, USA), supplemented with 10% FBS (12484-028; Invitrogen), 100 U of penicillin/100 µg of streptomycin (15140-122; Invitrogen) and 100 µM non-essential amino acids (11140-050; Invitrogen, Carlsbad, CA, USA), and grown in a humidified atmosphere containing 5% CO_2_ at 37°C. GC-1 cells were seeded at 20% of confluence and allowed to grow for 24 hours before treatment.

The monoamine oxidase inhibitor tranylcypromine (T) has been reported as a potent suppressor of the enzymatic activity of KDM1 [43], whereas trichostatin A (TSA) is a well characterized inhibitor of histone deacetylases [44]. In order to target KDM1 and HDAC1 with T and/or TSA, we examined the influence of these inhibitors on GC-1 cell viability, morphology and proliferation. Since trichostatin A is known to induce apoptosis and to affect cell cycle progression [45], [46], [47], GC-1 cells were exposed to varying TSA concentrations (25 nM, 50 nM, 100 nM, and 300 nM) to determine the optimal minimal dose which caused a reduction in histone H3 acetylation while having the least impact on cell viability. Treatment with 50 nM TSA was determined as most efficient – due to global changes in histone H3 acetylation levels without cytotoxicity (Figure S1). Cell viability was not altered by T concentrations ranging from 10 µM to 1 mM. A working concentration of 100 µM T was selected as this dose has been shown to inhibit KDM1 and cause epigenetic changes at the promoters of specific genes [43], [48]. As has been reported before [48], [49] no global changes in histone methylation occurred following treatment with T (Figures S1, S2). Medium was removed and cells were treated for 16 hours with either 100 µM T (13492-01-8, Biomol, Plymouth Meeting, PA, USA), or 50 nM TSA (T8552; Sigma-Aldrich, Saint Louis, MO, USA), or 0.000754% dimethylsulphoxide (DMSO) (D2650; Sigma-Aldrich), the solvent of TSA, or a cocktail of 100 µM T and 50 nM TSA. After 16 h of exposure, treated GC-1 cells were morphologically indistinguishable from control cells and cell counts after trypan blue staining revealed no differences in cell viability between treated and control cells.

### Western Blotting

Total protein of control or treated GC-1 cells were extracted with Laemmli buffer (62.5 mM Tris-HCl, pH 6.8/2% SDS/25% Glycerol/0.01% Bromophenol blue) containing 5% of β-Mercaptoethanol. Proteins were resolved by standard SDS-PAGE and electroblotted onto nitrocellulose membranes as previously described [35]. The membranes were incubated with the primary antibody followed by the corresponding horseradish peroxidase-conjugated secondary antibody (all antibodies are listed in Table S1). Beta-Actin was used for normalization of KDM1 and HDAC1 levels, whereas the total amount of histone H3 was used for normalization of Western blots for histone H3 modifications. Antibody signals were detected using SuperSignal West Pico Chemiluminescent Substrate (Thermo Scientific, Rockford, IL, USA). Band intensities were measured using the AlphaDigiDoc analysis system (Alpha Innotech Corporation, San Leandro, CA, USA). If not otherwise indicated each experiment was replicated a minimum of three times.

### Immunoprecipitation Assay

1×10∧6 GC-1 cells were harvested in 1 ml modified RIPA (50 mM Tris-HCl, pH 7.5/300 mM NaCl/1% NP-40/50 mM NaF (sodium fluoride)/1 mM PMSF/100 µM NaVO_3_/proteinase inhibitor cocktail (P8340; Sigma-Aldrich), sonicated (6×(5 sec on/5 sec off), amplitude: 25%) and kept on ice for 30 min. Soluble extracts were separated by centrifugation at 10000× g for 20 min. 250 µl of total cell extract were used for each IP or the corresponding control and each volume was made up to 500 µl with RIPA containing the full complement of inhibitors. Then, cell extracts were pre-cleared for 1 h on protein A-Agarose beads (Roche Diagnostics, Mannheim, Germany) and collected as supernatant after centrifugation. The supernatants were incubated over night at 4°C with either 5 µg of anti-HDAC1 (clone 2E10; Millipore, Billerica, MA, USA), or 5 µg of anti-KDM1 (Abcam, Cambridge, MA, USA) antibody, or the appropriate controls, which included mouse IgG and rabbit IgG (Jackson ImunoResearch Laboratories, West Grove, PA, USA). Protein A-Agarose beads were pre-adsorbed with 0.05% BSA prior to capturing the immune complex for 1 h at 4°C. The beads were then washed three times with RIPA buffer and captured proteins were eluted in SDS-PAGE sample buffer for Western blot analysis.

### Reverse transcription and real-time PCR

Total RNA was extracted using Purezol (BioRad, Hercules, CA, USA) according to the manufacturer's instructions. Then, 2 µg of total RNA was reverse transcribed using the High Capacity cDNA Reverse Transcription kit, which includes random primers (Applied Biosystems, Foster City, CA, USA). Primers were designed using Primer Express software (Applied Biosystems). Primer information is given in Table S2. PCR reactions were performed following the Power SYBR Green PCR Master Mix protocol (Applied Biosystems) with an ABI Prism 7500 apparatus (Applied Biosystems). The measured amount of each cDNA was normalized to *Gapdh*. In order to avoid pseudogene amplification, *Pou5f1* expression was additionally analyzed using the primer Mm00658129_gH *Pou5f1* TaqMan gene expression assay (Applied Biosystems) and the TaqMan Fast Universal PCR Master Mix (Applied Biosystems), following manufacturer's instructions. The 18S rRNA TaqMan gene expression assay (primer Hs99999901_s1, Applied Biosystems) was used for normalization. If not otherwise indicated, each experiment was repeated independently a minimum of three times, and each sample and the corresponding negative controls were run in triplicates.

### In silico promoter analysis

We performed an *in silico* analysis using the CSH TRED database [50], [51] (http://rulai.cshl.edu/cgibin/TRED/tred.cgi?process=home) to localize the transcriptional start sites (TSS) of *Gfra1* (*Gfra1* promoter ID 63194) and *Myod1* (*Myod1* promoter ID 76703), which were concordant with start sites of *Gfra1* and *Myod1* transcripts as given by ensemble genome browser (http://www.ensembl.org/index.html) and the UCSC genome browser (http://genome.ucsc.edu/index.html?org=Mouse&db=mm9&hgsid=125767812).

### Chromatin Immunoprecipitation (ChIP) assay followed by real-time PCR

ChIPs were performed following the optimized instructions to measure the levels of histone methylation and acetylation at targeted gene enhancer and promoter regions in GC-1 cells following treatment. Moreover, ChIP assays were used to confirm the presence of KDM1- and/or HDAC1- at *Pou5f1* and *Gfra1* gene promoters in GC-1 cells. Cells were rinsed with ice-cold PBS once and fixed in 1% formaldehyde for 10 min at room temperature to cross-link DNA and proteins. Glycine (0.125 M) was then added to quench the formaldehyde. Subsequently, the cells were rinsed twice in ice-cold PBS, scraped and collected by centrifugation at 4°C. Cells were then resuspended in 2 mL of FA lysis buffer (50 mM HEPES pH 7.5, 140 mM NaCl, 1 mM EDTA, 1% triton X-100, 0.1% sodium deoxycholate 10%, 0.1% SDS and protease inhibitor cocktail (P8340; Sigma-Aldrich). The chromatin was sheared by sonication to an average fragment size of 500 to 1000 bp. 50 µL of sample was removed to serve as input control, whereas 50 µg of protein was used for each immunoprecipitation, including IgG and serum controls. After a dilution 1∶10 of each sample with RIPA buffer (50 mM Tris-HCl pH 8, 150 mM NaCl, 2 mM EDTA ph8, 1% NP-40, 0.5% sodium deoxycholate, 0.1%SDS and protease inhibitors), antibodies were added to the corresponding sample (find detailed antibody information in Table S1). 20 µL of protein A-Agarose beads (pre-absorbed with BSA (NEB, Pickering, Ontario, Canada) and sonicated single stranded herring sperm DNA (ab46666, Abcam) was added to all samples that were then rotated overnight at 4°C. After three washings with a buffer containing 0.1% SDS/1% Triton X-100/2 mM EDTA pH 8/150 mM NaCl/20 mM Tris-HCl pH 8 and one with a final wash buffer (0.1% SDS, 1% Triton X-100, 2 mM EDTA pH 8, 500 mM NaCl, 20 mM Tris-HCl pH 8) the chromatin-antibody complexes were eluted and the DNA-proteins cross-links were reversed for all the samples (including the input) at 65°C overnight in presence of 1 µg of RNase A (Sigma-Aldrich). Genomic DNA was recovered and purified with the QIAquick PCR purification kit (Qiagen, Hilden, Germany). Quantitative real-time PCR amplifications were performed using primers specific for *Pou5f1* and *Gfra1* promoters, or *Gapdh* control primers (Table S2), which were designed using Primer Express Software (Applied Biosystems). Primers spanned regions −2281 to −2212 and −854 to −781 relative to the *Pou5f1* transcription start site, and −1071 to −997 and +104 to +179 relative to the putative *Gfra1* transcription start site. In order to analyze a putative promoter occupancy of KDM1- and/or HDAC1-containig protein complexes approximately 3000 bp of *Pou5f1* and *Gfra1* promoter regions were screened by ChIP-qPCR for KDM1- and/or HDAC1 abundance. Power SYBR Green PCR Master Mix (Applied Biosystems) was used with an ABI Prism 7500 apparatus (Applied Biosystems) following manufacturer's instructions. Relative quantification of the genomic DNA received by ChIP was performed with the comparative C_T_ method. Briefly, after confirmation of the PCR efficiency for each primer pair used in this study (Table S2), the ΔC_T_ values were determined by subtracting the average C_T_ value of the normalized input from the average C_T_ value of the corresponding IP samples or the IgG and normal serum controls, respectively. In order to determine the fold change of target DNA in treated GC-1 cells over target DNA in DMSO controls, the ΔΔC_T_ was calculated by subtracting the ΔC_T_ of the DMSO from the ΔC_T_ of TSA or TSA and T treated cells. The amount of target DNA is finally given by the formula 2∧-(ΔΔC_T_).

One experiment comprised the following treatments: cells exposed to DMSO, or TSA, or a cocktail of T and TSA. For one experiment two independent plates per treatment have been pooled. Each ChIP was performed on three independent experiments.

### Pyrosequencing

CpG islands in *Pou5f1* or *Gfra1* promoter regions were identified with ”The sequence manipulation suite” database (http://www.bioinformatics.org/sms/index.html) [52] using the method described by Gardiner-Garden and Frommer [53]. Promoter methylation was determined by pyrosequencing for allele quantification (PSQ H96A, Biotage, Sweden), which is a real-time sequencing-based DNA analysis that quantifies multiple and consecutive CpG sites individually. Briefly 1000 ng of sample DNA was bisulfite treated using the Zymo DNA Methylation Kit (Zymo research, Orange, CA, USA). Bisulfite treated DNA was eluted in 10 µl volume and 1 µl of it is used for each PCR. PCR was performed using 10X PCR buffer, 3.0 mM MgCl2, 200 µM of each dNTP, 0.2 µM each of forward and reverse primers, HotStar DNA polymerase (Qiagen) 1.25 U, and ∼10 ng of bisulfite converted DNA per 50 µl reaction. PCR cycling conditions were: 94°C 15 min; 45 cycles of 94°C 30 s; 53°C 30 s; 72°C 30 s; 72°C 5 min; and then products were held at 4°C. The PCR was performed with one of the PCR primers biotinylated to convert the PCR product to single-stranded DNA templates. The PCR products (each 10 µl) were sequenced by Pyrosequencing PSQ96 HS System (Biotage AB) following the manufacturer's instructions (Biotage, Kungsgatan, Sweden). The methylation status of each locus was analyzed individually as a T/C SNP using QCpG software (Biotage, Kungsgatan, Sweden). Assay design and pyrosequencing sequencing was performed by EpigenDX (Worcester, MA, USA). The methylation assay ASY585 covered the region -190 to +26 relative to the *Pou5f1* CpG transcriptional start site and the ADS932 assay comprised the region +26 to +143 relative to the putative *Gfra1* transcriptional start site.

### Statistical analyses

If not otherwise indicated, all experiments were repeated at least three times. All values are expressed as means ± SEM. To detect significant effects of treatments a one-way analysis of variance (ANOVA) was performed and to identify differences between means the Student-Newman-Keuls test was applied.

## Results

### The KDM1/HDAC1 complex is present in cultured GC-1

**Figure 1:**
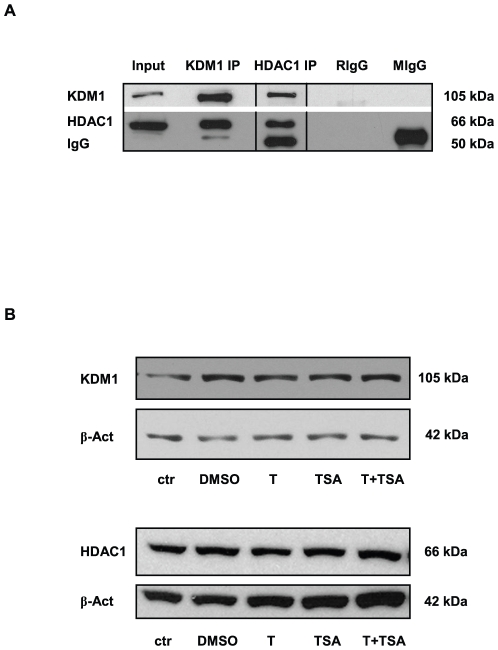
KDM1 and HDAC1 form heteromeric complexes in GC-1 cells. (**A**) Endogenous KDM1 and HDAC1 were co-immunoprecipitated with specific antibodies against KDM1 and HDAC1. Total GC-1 protein extract was used as input control. Immunoglobulin G (IgG), Rabbit IgG (RIgG), mouse IgG (MIgG). (**B**) Western blot analyses of HDAC1 and KDM1 in protein extracts isolated from GC-1 cells grown in culture medium (control, ctr), or in culture medium containing either dimethylsulphoxide (DMSO; TSA solvent), or tranylcypromine (T), or trichostatin A (TSA), or tranylcypromine and TSA (T+TSA). Western blots were reprobed with an antibody against beta-Actin as a loading control. Treatment of GC-1 cells with tranylcypromine and TSA does not alter protein levels of KDM1 and HDAC1.

*In vivo* co-immunoprecipitation experiments on total GC-1 protein extracts demonstrated that in this cell line as in the testis KDM1 is associated with HDAC1 (Figure 1A). To determine whether targeting of the KDM1/HDAC1 repressor complex by T and/or TSA altered KDM1 or HDAC1 expression or protein stability, Western blot analysis was performed on total protein extracts isolated from GC-1 cells grown in regular culture medium, or culture medium supplemented with either T, TSA, DMSO (the solvent of TSA), or T and TSA. Treatment did not alter KDM1 or HDAC1 protein levels indicating that targeting the enzymatic activities of KDM1 and HDAC1, either individually or combined, does not influence their expression (Figure 1B).

### Identification of spermatogenic genes sensitive to regulation by histone methylation and acetylation by treatment with trichostatin A and/or tranylcypromine

**Figure 2:**
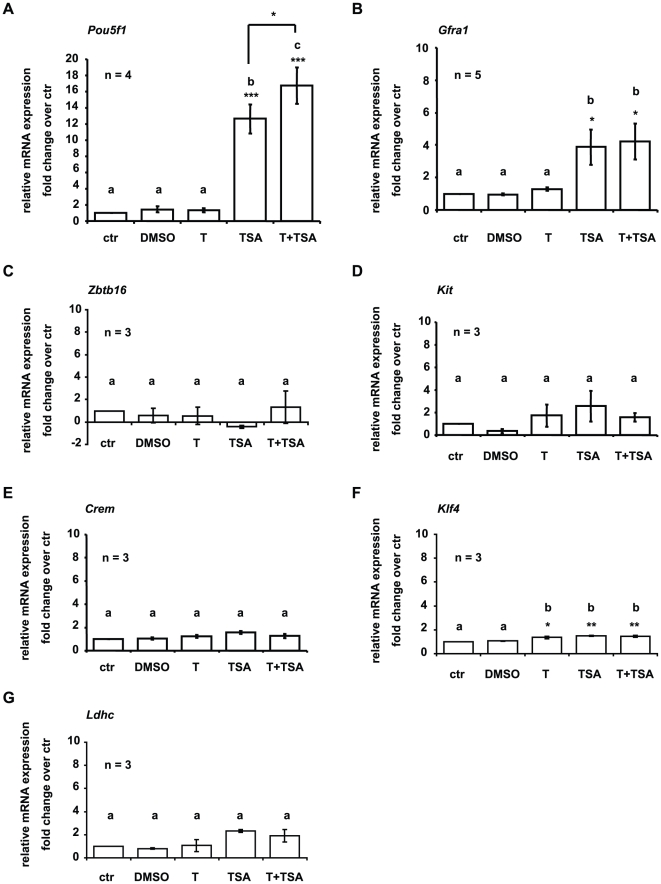
Treatment induces Pou5f1 and Gfra1 gene expression in GC-1 cells. QPCR analyses of spermatogenic gene expression in GC-1 cells, either grown in culture medium (control, ctr), or treated with tranylcypromine (T), or trichostatin A (TSA), or dimethylsulphoxid (DMSO; TSA solvent), or tranylcypromine and TSA (T+TSA). To avoid detection of expressed pseudogenes a TaqMan assay was performed to analyze *Pou5f1* gene expression. *Pou5f1* signals were normalized to 18S rRNA. All other genes were analyzed using SYBR green and signals were normalized to *Gapdh*. Values shown on the graphs are based on the change in expression in treated cells compared to the level of expression in control cells whereby the level of expression in control cells was defined as one. Thereby the fold change in expression indicates the ratio of normalized target gene expression in treated cells over normalized target gene expression in control cells. Data are expressed as mean ± SEM; *p<0.05, **p<0.01, ***p<0.001. Means with different letters are significantly different. N indicates the number of independent experiments, and per experiment each sample and the corresponding negative controls were run in triplicates. Trichostatin A treatment triggers *Pou5f1* (**A**) and *Gfra1* (**B**) gene expression in GC-1 cells. Treatment causes a slight but significant increase in Krueppel-like factor 4 (*Klf4*) (**F**) expression. Zinc finger and BTB domain containing 16 (*Zbtb16*) (**C**), kit oncogene (*Kit*) (**D**), cAMP responsive element modulator (*Crem*) (**E**), and lactate dehydrogenase C (*Ldhc*) (**G**) are not affected by treatment.

In order to identify target genes sensitive to the drug treatment we selected seven genes that have been shown to be expressed at defined stages of spermatogenesis. The *Myod1* gene, which is not expressed in germ cells, was used as control for specificity of treatment to ensure that the drug treatments were not creating an overall more open chromatin state. Under control conditions we detected very low to no expression of the following spermatogonia markers (Table S3): *Pou5f1* [6], [54], [55], zinc finger and BTB domain containing 16 (*Zbtb16*) [3], [54], or kit oncogene (*Kit*), [56], [57], [58], [59], [60]. However, *Gfra1* [4], [61], [62], [63], [64] was weakly expressed in untreated GC-1 cells (Table S3). On the other hand transcripts of the following genes known to be expressed in spermatocytes were found under control conditions (Table S3): cAMP responsive element modulator (*Crem*) [65], [66] and in agreement with Hofmann and co-workers (1992) [42] low levels of testicular lactate dehydrogenase-C4 isozyme (*Ldhc*) [67]. Interestingly, Krueppel-like factor 4 (*Klf4*) is also expressed in untreated GC-1 cells (Table S3). In the mouse testis, *Klf4* is strongly abundant in round spermatids [68], [69], but it is also found at low levels in cultured spermatogonial stem cells [70]. Remarkably, treatment of GC-1 cells with TSA or TSA in combination with T induced the expression of spermatogonia cell markers *Pou5f1* (TSA induced a 12.6-fold increase, Figure 2A) and *Gfra1* (TSA, or TSA and T combined induced a 4-fold increase, Figure 2B) and the maximal expression of *Pou5f1* was induced by a combined treatment of TSA and T (16.8-fold increase, Figure 2A). There was no induction of *Pou5f1* or *Gfra1* expression when GC-1 cells were exposed to DMSO or T alone (Figure 2A,B). Treatment with TSA significantly increased expression of *Gfra1* (4-fold increase) but combined treatment with TSA and T did not differ from TSA alone (Figure 2B). No induction or alterations in gene expression of the other marker genes were observed (Figure 2C–E,G) with the exception of *Klf4* where a small but significant increase was detected following treatment with T (1.4-fold increase) or TSA alone (1.5-fold increase) and T and TSA combined (1.5-fold increase) (Figure 2F). (Ct values of genes analyzed in untreated GC-1 cells are summarized in Table S3. Changes in gene expression due to treatment are depicted as fold change over control conditions (untreated GC-1 cells) in Figure 2.)

### Trichostatin A treatment alters the epigenetic landscape at Pou5f1 and Gfra1 promoters

**Figure 3:**
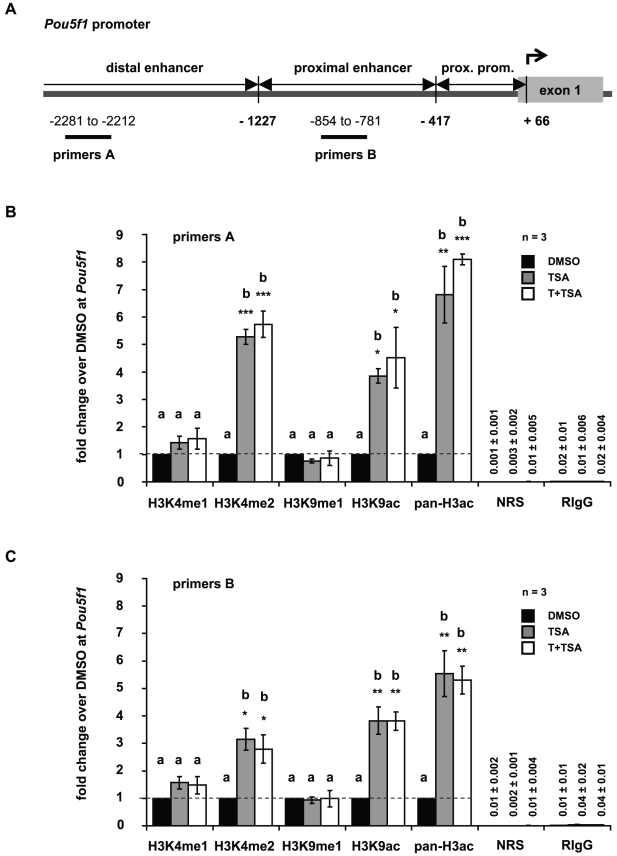
Tranylcypromine and trichostatin A treatment influences posttranslational modifications of histone H3 at Pou5f1 promoter. (**A**) Schematic representation of the *Pou5f1* promoter. Numbers depict the positions of primer pairs used for ChIP-qPCR relative to the corresponding transcriptional start site. (**B,C**) Analyses of histone H3 methylation and acetylation levels in the *Pou5f1* promoter by ChIP followed by qPCR in GC-1 cells that have been cultured in the presence of dimethylsulphoxide (DMSO, control, black bars), or trichostatin A (TSA, grey bars), or both inhibitors, tranylcypromine and TSA (T+TSA, white bars). Signals of target DNA received from DMSO control cells served as base and were defined as one. Fold change indicates 2∧-(ΔΔC_T_) of target DNA in treated cells over 2∧-(ΔΔC_T_) of target DNA in DMSO controls (Y-axis). Epigenetic modifications are depicted on the X-axis. Data are expressed as mean ± SEM; *p<0.05, **p<0.01, ***p<0.001. Means with different letters are significantly different. N indicates the number of independent experiments, and per experiment each sample and the corresponding negative controls were run in triplicates.

**Figure 4:**
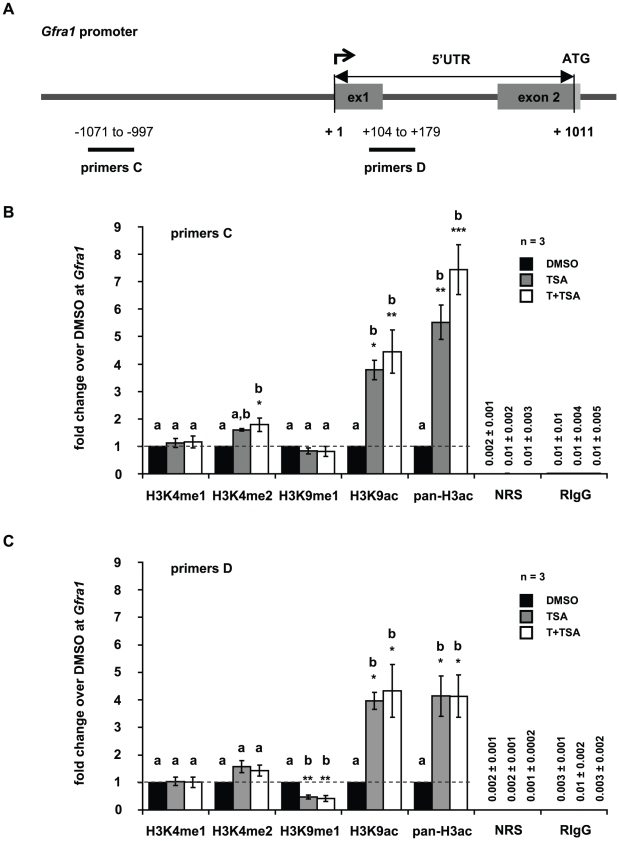
Tranylcypromine and trichostatin A treatment influences posttranslational modifications of histone H3 at Gfra1 promoter. (**A**) Schematic representation of the *Gfra1* promoter. Numbers depict the positions of primer pairs used for ChIP-qPCR relative to the corresponding transcriptional start site. (**B,C**) Analyses of histone H3 methylation and acetylation levels in the *Gfra1* promoter by ChIP and qPCR in GC-1 cells that have been cultured in the presence of dimethylsulphoxide (DMSO, control, black bars), or trichostatin A (TSA, grey bars), or both inhibitors, tranylcypromine and TSA (T+TSA, white bars). Signals of target DNA received from DMSO control cells served as base and were defined as one. Fold change indicates 2∧-(ΔΔC_T_) of target DNA in treated cells over 2∧-(ΔΔC_T_) of target DNA in DMSO controls (Y-axis). Epigenetic modifications are depicted on the X-axis. Data are expressed as mean ± SEM; *p<0.05, **p<0.01, ***p<0.001. Means with different letters are significantly different. N indicates the number of independent experiments, and per experiment each sample and the corresponding negative controls were run in triplicates.

In all experiments performed in this study no differences were observed between GC-1 cells grown in culture medium and GC-1 cells exposed to DMSO. As the treatment with T alone did not induce changes in gene expression (Figure 2) or alter global histone methylation or acetylation levels (Figures S1, S2), we focused on TSA and the combined treatment of TSA and T in subsequent experiments to determine gene-specific alterations in histone methylation, histone acetylation, and DNA methylation at *Pou5f1* and *Gfra1* gene promoters. GC-1 cells grown in culture medium supplemented with DMSO were used as the control. To correlate epigenetic changes at these promoters with changes in gene expression, we performed chromatin immunoprecipitation (ChIP) followed by quantitative real-time PCR. The mouse *Pou5f1* promoter has been well characterized and comprises defined regulatory regions (distal enhancer, proximal enhancer, and proximal promoter) that are critical for its activation [71], [72]. To cover the regulatory regions of the *Pou5f1* promoter and a broad region of the *Gfra1* promoter, ChIP-qPCR primers were positioned at intervals of approximately 1000 bp as depicted in Figures 3A and 4A, respectively. Since DNA fragments of 500–1000 bp were generated with sonication, epigenetic changes in the distal enhancer region of the *Pou5f1* promoter were detected with primer pair A (−2281 to −2212 relative to TSS), and changes in the proximal enhancer/proximal promoter region with primer pair B (−854 to −781 relative to TSS) (Figure 3A). In order to identify epigenetic changes in the *Gfra1* promoter, primer pair C (−1071 to −997 relative to TSS) and D (located in the 5′UTR of *Gfra1* at +104 to +1011 relative to TSS) were used in qPCR analyses (Figure 4A).

Using ChIP-qPCR we compared the levels of H3K4 mono- and dimethylation, H3K9 monomethylation, H3K9 acetylation, and pan-H3 acetylation at the promoters of *Pou5f1* and *Gfra1* between cells treated with either TSA, or TSA and T, or the DMSO control. Treatment of GC-1 cells with TSA, or TSA in combination with T significantly increased gene activating histone H3K4 dimethylation, H3K9 acetylation, and pan-H3 acetylation marks in the distal enhancer and the proximal enhancer/proximal promoter region of *Pou5f1*, whereas levels of H3K4 monomethylation and H3K9 monomethylation were indistinguishable from DMSO controls (Figure 3B,C). No enhanced effect on histone H3K4 methylation was found by treating with TSA in combination with T.

Analyzing the epigenetic signature of the *Gfra1* promoter region after TSA exposure or the combined treatment of TSA and T by ChIP-qPCR, we identified a significant increase in activating H3K9 acetylation and pan-H3 acetylation marks in the putative promoter region upstream of the TSS and downstream in the 5′UTR of *Gfra1* (Figure 4B,C). A slight (approximately twofold) but significant increase in H3K4 dimethylation was observed upstream of the *Gfra1* TSS using the combined treatment of TSA and T. There were no alterations in either H3K4 monomethylation or H3K9 monomethyaltion in this region (Figure 4B). On the contrary, we identified a slight but significant decrease in the repressive H3K9 monomethylation mark in the 5′UTR of *Gfra1* using TSA alone or in combination with T, but H3K4 mono- and dimethylation levels were indistinguishable from the DMSO controls (Figure 4C). Here, no enhanced effect on histone H3K4- or H3K9 methylation was found by treating the cells with TSA in combination with T (Figure 4C).

### Effect of tranylcypromine and trichostatin A on gene expression and the epigenetic landscape of Myod1

**Figure 5:**
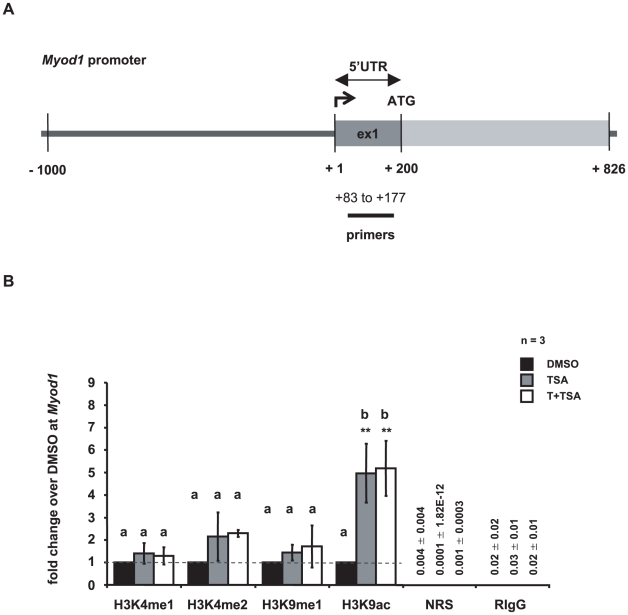
Histone H3 methylation at Myod1 promoter is not affected by tranylcypromine and trichostatin A treatment. (**A**) Schematic representation of the *Myod1* promoter. Numbers depict the positions of primers used for ChIP-qPCR relative to the corresponding transcriptional start site. (**B**) ChIP analyses followed by qPCR reveal changes in the epigenetic landscape of the *Myod1* promoter upon treatment. Despite the increase in H3K9 acetylation, *Myod1* is not expressed in untreated GC-1 cells nor induced as a consequence of treatment, as shown in Table S4. Depicted are histone H3 methylation and acetylation levels in GC-1 cells that have been cultured in the presence of dimethylsulphoxide (DMSO, control, black bars), or trichostatin A (TSA, grey bars), or both inhibitors, tranylcypromine and TSA (T+TSA, white bars). Signals of target DNA received from DMSO control cells served as base and were defined as one. Fold change indicates 2∧-(ΔΔC_T_) of target DNA in treated cells over 2∧-(ΔΔC_T_) of target DNA in DMSO controls (Y-axis). Epigenetic modifications are depicted on the X-axis. Data are expressed as mean ± Stdev; *p<0.05, **p<0.01. Means with different letters are significantly different. N indicates the number of independent experiments, and per experiment each sample and the corresponding negative controls were run in triplicates.

To demonstrate specificity of the effects on the expression of *Pou5f1* and *Gfra1* following treatment we examined the expression and epigenetic modifications of myogenic differentiation 1 (*Myod1*), which is important for muscle development (for review see [73]) and not expressed in GC-1 cells under control conditions (Table S4). ChIP analysis of the *Myod1* promoter showed that treatment did not alter H3K4 mono- and dimethylation or H3K9 methylation. Treatment with TSA or T and TSA increased H3K9 acetylation (Figure 5). However these changes in acetylation alone did not induce *Myod1* expression (Table S4).

### Effect of tranylcypromine and trichostatin A on DNA methylation of CpG islands in Pou5f1 and Gfra1 promoters

**Figure 6:**
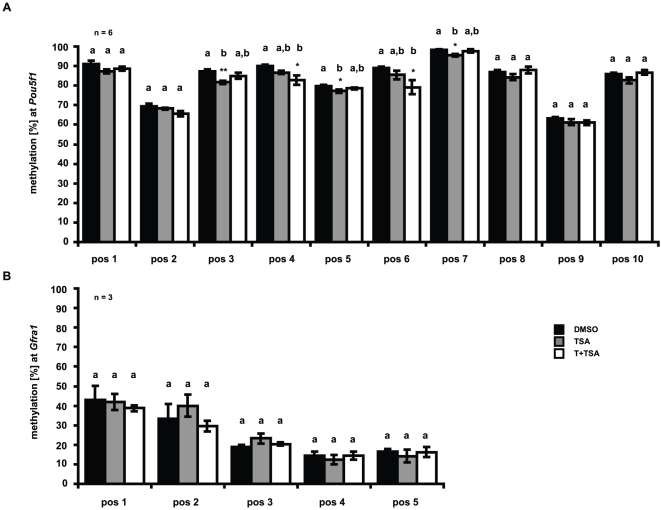
Effect of tranylcypromine and trichostatin A treatment on CpG methylation at Pou5f1 and Gfra1 promoters. DNA was extracted, treated with bisulfite, amplified by PCR and pyrosequencing was used to determine the bisulfite-converted sequence of CpG sites in (**A**) *Pou5f1* (positions −190 to +26 relative to the transcriptional start site) and (**B**) *Gfra1* promoters (positions +26 to +143 relative to the transcriptional start site). The methylation status of each position is depicted as the mean percentage of “n” independent experiments ± SEM; *p<0.05, **p<0.01. Means with different letters are significantly different. Black bars indicate DMSO controls, grey bars depict TSA treated samples and white bars represent samples that have been exposed to both inhibitors, tranylcypromine and TSA (T+TSA).

It is well known that histone methylation and DNA CpG methylation are functionally linked [49], [74], [75], [76], [77], [78]. We performed bisulfite treatment followed by pyrosequencing to determine the effect of TSA and T on the DNA methylation status of *Pou5f1* and *Gfra1* promoters. Primers used in these assays were located in the proximal promoter of *Pou5f1* (−190 to +26 relative to TSS) and *Gfra1* (+26 to +143 relative to TSS), overlapping or in closed vicinity with regions analyzed by ChIP-qPCR, which allowed us to relate the DNA methylation status of the promoter to the histone methylation and acetylation status and thus changes in gene expression between treated GC-1 cells and DMSO controls. Ten CpG dinucleotide positions were assayed for methylation in the defined region of the *Pou5f1* promoter and identified as hypermethylated (>70% methylation) in all cells, comprising DMSO controls, or TSA-, or TSA and T treated GC-1 cells (Figure 6A). Interestingly, depending on the position of the dinucleotide and the treatment, slight but significant reductions in CpG methylation could be observed in the assayed *Pou5f1* promoter region (Figure 6A). On the contrary, CpG dinucleotides of the assayed *Gfra1* promoter region are slightly methylated as indicated in Figure 6B. Here, we were not able to detect any significant changes occurred between treated cells and controls.

### KDM1 and HDAC1 occupy Pou5f1 and Gfra1 promoters in GC-1 cells

**Figure 7:**
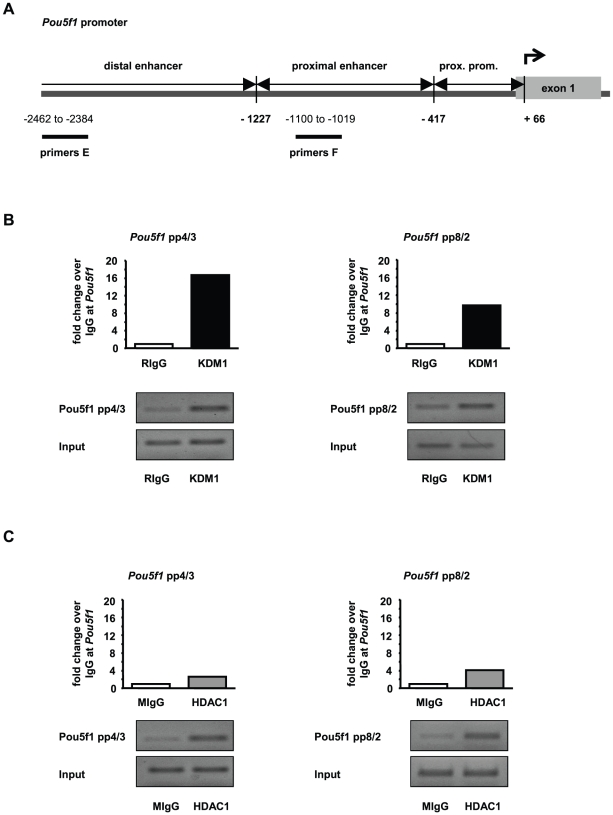
KDM1- and HDAC1-containing protein complexes are located at the Pou5f1 promoter in GC-1 cells. (**A**) Schematic representation of the *Pou5f1* promoter. Numbers depict the positions of primer pairs used for ChIP-qPCR relative to the corresponding transcriptional start site. (**B,C**) ChIP-qPCRs demonstrate occupancy of the *Pou5f1* promoter by KDM1- (**B**) and HDAC1-containing protein complexes (**C**). ChIP assays were performed on GC-1 cells that have been cultured in the presence of dimethylsulphoxide using either a specific antibody (recognizing either KDM1 (black bars) or HDAC1 (grey bars)) or the corresponding IgG controls (white bars), (rabbit IgG  =  RIgG; mouse IgG  =  MIgG). Each bar represents the ChIP-qPCR results of two independent, pooled experiments. Signals of target DNA received from IgG controls served as base and were defined as one. Fold change represents the 2∧-(ΔΔC_T_) value, where the normalized IgG signal is subtracted from the normalized signal of the specific antibody. QPCR results were analyzed on an agarose gel and shown underneath the corresponding graphs.

**Figure 8:**
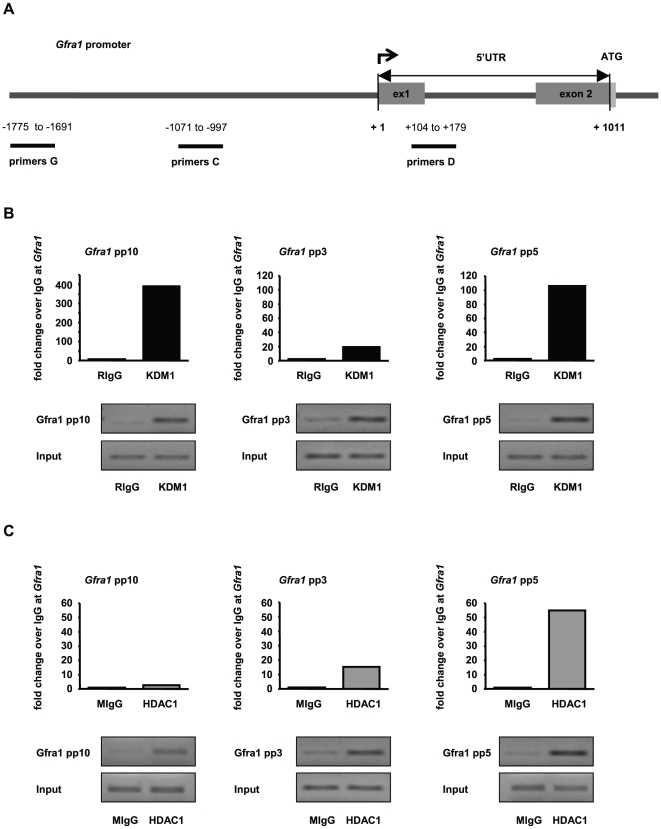
KDM1- and HDAC1-containing protein complexes are located at the Gfra1 promoter in GC-1 cells. (**A**) Schematic representation of the *Gfra1* promoter. Numbers depict the positions of primer pairs used for ChIP-qPCR relative to the corresponding transcriptional start site. (**B,C**) ChIP-qPCRs demonstrate occupancy of the *Gfra1* promoter by KDM1- (**B**) and HDAC1-containing protein complexes (**C**). ChIP assays were performed on GC-1 cells that have been cultured in the presence of dimethylsulphoxide using either a specific antibody (recognizing either KDM1 (black bars) or HDAC1 (grey bars)) or the corresponding IgG controls (white bars), (rabbit IgG  =  RIgG; mouse IgG  =  MIgG). Each bar represents the ChIP-qPCR results of two independent, pooled experiments. Signals of target DNA received from IgG controls served as base and were defined as one. Fold change represents the 2∧-(ΔΔC_T_) value, where the normalized IgG signal is subtracted from the normalized signal of the specific antibody. QPCR results were analyzed on an agarose gel and shown underneath the corresponding graph.

ChIP-QPCR was used to confirm that the protein targets of T and TSA, namely KDM1 and/or HDAC1 interact with *Pou5f1* and *Gfra1* gene promoters. These assays demonstrated a 17-fold enrichment of KDM1 in the distal enhancer and a 10-fold enrichment of KDM1 in the proximal enhancer of the *Pou5f1* promoter in GC-1 cells (Figure 7B). HDAC1 was found to be enriched at the *Pou5f1* promoter, namely 3-fold in the distal and 4-fold in the proximal enhancer region (Figure 7C). Analyzing KDM1 and HDAC1 occupancy of the *Gfra1* promoter in GC-1 cells, we were able to detect a 390-fold enrichment of KDM1 in the distal (−1775 relative to TSS) and a 20-fold enrichment in the more proximal promoter region (−1071 relative to TSS). Moreover KDM1 was found to be 106-times enriched in the 5′UTR of the Gfra1 promoter (Figure 8B). HDAC1 was 3-times enriched in the distal Gfra1 promoter region, an over 15-fold enrichment of HDAC1 was detectable in the proximal promoter region and a 55-fold enrichment in the 5′UTR of the Gfra1 promoter (Figure 8C). According to a BLAST search at NCBI (http://blast.ncbi.nlm.nih.gov/) the antibody used to detect HDAC1 may have some cross-reactivity with two isoforms of HDAC1 (XP_001480415.1, XP_001480417.1) which would impact the results by diluting the signal at the target regions. These results strongly suggest that KDM1 and HDAC1 occupy *Pou5f1* and *Gfra1* promoters in GC-1 cells and function in the regulation of expression.

## Discussion

**Figure 9:**
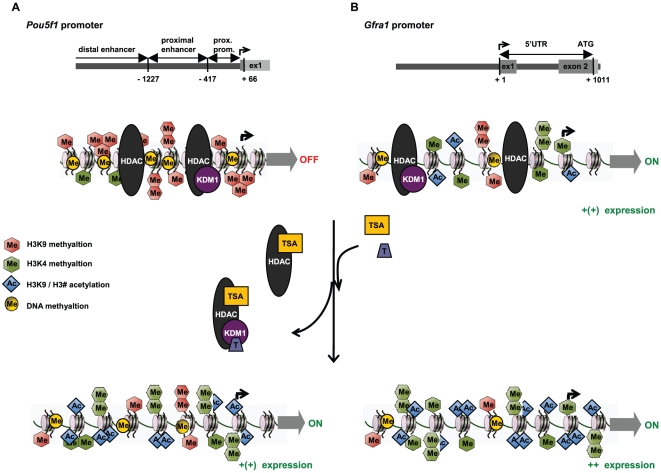
Epigenetic remodeling in Pouf5f1 and Gfra1 promoter regions - a proposed model to explain changes in gene expression in GC-1 cells after Tranylcypromine and TSA treatment. (**A**) In untreated GC-1 cells (regular growth conditions), repressive epigenetic marks (e.g. H3K9 methylation and CpG methylation) keep the *Pou5f1* promoter silent, by creating a tight chromatin structure and preventing the binding of the transcription machinery. Multi-protein complexes that contain writers of repressive epigenetic marks, e.g. HDACs and/or HDAC/KDM1, create a repressive chromatin state. Blocking these enzymes using TSA (targets HDACs) and/or tranylcypromine (represses KDM1) results in the accumulation of activating epigenetic marks (H3K4 methylation, H3 acetylation), thereby making it accessible for transcription factors and the transcription machinery, which eventually results in the induction of *Pou5f1* expression. (**B**) Repressive and activating epigenetic marks at the promoter are responsible for the weak expression of *Gfra1* in untreated GC-1 cells. The exposure of GC-1 cells to tranylcypromine and TSA blocks the gene silencing activities of HDACs and/or HDAC/KDM1 and allows for the accumulation of activating epigenetic marks. Thus the treatment shifts the epigenetic landscape at the *Gfra1* promoter to a more active state and eventually triggers a stronger transcription of *Gfra1*. Histone deacetylase (HDAC), lysine-specific demethylase 1 (KDM1), tranylcypromine (T), trichostatin A (TSA).

In this study we revealed epigenetic mechanisms that are likely to be involved in the expression of key genes in spermatogonia, namely *Pou5f1* and *Gfra1*. These experiments implicate histone H3 methylation and acetylation in the regulation of their expression and suggest that transcription is influenced by HDACs and KDM1. The underlying molecular mechanisms revealed by pharmacological treatments of the GC-1 cell line suggest that in spermatogonia expression of *Pou5f1* and *Gfra1* is maintained by an enrichment of histone H3 acetylation and dimethylation of histone H3 at lysine 4. Based on our results we suggest that as spermatogonia differentiate the loss of expression of *Pou5f1* and *Gfra1* may be concomitant with gene-specific activity of HDACs and KDM1 and the consequent removal of gene activating histone H3 acetylation and H3K4 dimethylation. These epigenetic changes are summarized in a model shown in Figure 9.

Histone acetylation is intimately involved in the epigenetic regulation of transcription and at a single gene locus a fine balance of histone acetyltransferase and deacetylase activity controls gene expression [79], [80], [81]. Histone hyperacetylation is associated with an open chromatin state and general transcriptional activity [10], [11], [12], [13], [14]. Inhibition of histone deacetylase activity by TSA has many faces: it can for instance result in de-differentiation of primordial germ cells into embryonic germ cells [82], accelerate murine stem cell differentiation [83], force cancer cells into a differentiated state [84], or in concert with a DNA methyltransferase inhibitor convert neurosphere cells into neurosphere-derived haematopoietic cells [85]. While we did not observe changes in cell morphology or behavior to indicate the GC-1 cell line altered its differentiation state, we did observe specific changes in gene expression that were associated to gene-specific changes in the epigenetic profile. In the brief time-scale of these experiments changes in differentiation would not be expected. For example in the case of induced pluripotent stem cells, virally transduced pluripotency factors (e.g. *Pou5f1*, *Klf4*, *Sox2*) need to be expressed for approximately twelve to eighteen days to initiate de-differentiation and reprogramming of mouse embryonic fibroblast cells [86].

Interestingly TSA treatment of the GC-1 cell line turned on specific genes expressed in spermatogonia as we saw an activation of spermatogonia cell markers *Gfra1* and *Pou5f1*. The lack of change in expression in the panel of genes examined suggests that expression of *Pou5f1* and *Gfra1 i*s a consequence of gene-specific epigenetic effects of the HDAC and KDM1 inhibitors and not due to global changes to the epigenome. In the present study, induced *Pou5f1* expression was associated with increased H3K4 dimethylation, H3K9 acetylation and pan-H3 acetylation at regulatory regions of the mouse *Pou5f1* promoter. Although we demonstrated that *Pou5f1* expression was maximal under the co-suppression of KDM1 and HDAC1, a closer look at the epigenetic marks on the promoter revealed that co-inhibition of HDAC and KDM1 did not further enhance gene activating H3K4 methylation marks. Therefore the enhanced gene activation under KDM1/HDAC1 inhibition may be a consequence of indirect KDM1/HDAC1 signaling. We postulate that the inhibition by TSA on HDACs and the consequent increased H3K4 dimethylation was due to an indirect effect on KDM1 since TSA does not target demethylases, but specifically inhibits histone deacetylases by binding to the catalytic core [87]. Evidence supporting this potential molecular mechanism comes from experiments showing that hyperacetylation of lysine residues on histone H3 abolished or attenuated KDM1 function, and that acetyl groups had to be removed before H3K4 demethylation by KDM1 occurred [88], [89]. Moreover we detected KDM1 and HDAC at our target genes promoters suggesting that effects are a consequence of drug action on the KDM1/HDAC complex.

The gain in histone H3K4 dimethylation at the *Pou5f1* promoter and associated gene activation we report here following HDAC inhibition and under the combined suppression of KDM1/HDAC is similar to what has been observed in embryonal carcinoma cells [43]. However, unlike the GC-1 cells, embryonal carcinoma cells increased *Pou5f1* expression when KDM1 was inhibited by T alone [43]. The differences in cellular response to T between the embryonal carcinoma cells and GC-1 cell used here may be due to differences in sensitivity to inhibition of KDM1 by T. Unlike GC-1 cells, embryonal carcinoma cells express high levels of KDM1 and have detectable levels of *Pou5f1* under control conditions [43].

Like *Pou5f1*, the expression of *Gfra1* in GC-1 cells seemed to be directed principally by HDACs since treatment with TSA caused a strong increase in activating H3K9- and total H3 acetylation in the *Gfra1* promoter region and consequent gene expression. In contrast to the major changes in acetylation only a slight increase in gene activating H3K4 dimethylation under the combined inhibition of KDM1/HDAC1 by TSA and T occurred, and this was not associated with enhanced expression in comparison to TSA alone. In this study there was no effect of inhibition of KDM1 by T alone on global histone modifications or changes in gene expression. Rather, treatment with T was effective only when used in combination with TSA. These effects are in line with previous studies where it has been reported that there was a lack of globally increased histone H3 methylation after genetic ablation of KDM1 in murine ES cells [49], and following the suppression of KDM1 by T, or its knockdown by siRNA in human cervical cancer cell lines [48]. Taken together these findings suggest that KDM1 activities are highly gene-specific and likely to involve its function in a protein complex with HDAC and other proteins.

At some gene promoters there is crosstalk between histone modifications and DNA methylation. In a previous study Ou and coworkers associated a TSA induced increase of histone acetylation, with a significant decrease in global DNA methylation [90]. It was thereby pertinent to assess the DNA methylation status at the promoter regions of *Pou5f1* and *Gfra1*. Overall no significant changes in DNA methylation occurred in either the *Pou5f1* or *Gfra1* promoter indicating that histone methylation and acetylation are the principles means of regulating gene expression at these promoters in GC-1 cells. However, depending on the treatment and the position of the CpG dinucleotides we observed a slight but significant decrease in DNA methylation in the analyzed *Pou5f1* promoter region, but none in the *Gfra1* promoter. We cannot completely rule out that the changes in DNA methylation observed in the *Pou5f1* promoter had an impact on the induction of its expression in treated GC-1 cells. But this seems to be unlikely as >60 to 70% methylation remained following treatment at the *Pou5f1* promoter and low methylation was found on the *Gfra1* promoter.

As the mechanisms and interpretation discussed here are based on a transformed spermatocyte-like cell line, this underlies the extrapolation to events *in vivo*. The use of an immortalized cell line such as the GC-1 holds certain limitations but the positive factors outweighed the negatives when choosing a model system and examining the alternatives. The GC-1 cells represent a relatively homogenous cell line that can be readily and robustly expanded without being co-cultured with somatic cells, while sustaining germ cell characteristics [42]. Moreover they are easy to manipulate and deliver an unlimited source of material, which is critical for ChIP experiments, which require millions of cells per treatment for analysis.

Importantly this study has revealed epigenetic mechanisms likely to be involved in the complex gene regulation in spermatogenesis. Worth considering is that environmental induced epigenetic mutations in developing germ cells may have consequences for lifetime fertility as there is likely to be plasticity in histone modifications.

## Supporting Information

Table S1 Antibodies used for Western blotting (WB) and chromatin-immunoprecipitation (ChIP).
(0.04 MB DOC)

Table S2 Primers and TaqMan gene expression assays used for qPCR or ChIP-qPCR.
(0.07 MB DOC)

Table S3 qPCR analysis of male germ cell marker gene expression in untreated GC-1 cells.
(0.04 MB DOC)

Table S4 qPCR analysis of MyoD gene expression in untreated and treated GC-1 cells.
(0.03 MB DOC)

Figure S1 Effect of tranylcypromine and trichostatin A on global Histone H3K4 methylation and H3 acetylation levels. Western blot analyses of global histone (A) H3K9 acetylation, (B) pan-H3 acetylation, (C) H3K4 monomethylation, and (D) H3K4 dimethylation on protein extracts isolated from GC-1 cells either grown in culture medium (control, ctr), or treated with tranylcypromine (T), trichostatin A (TSA), or dimethylsulphoxid (DMSO; TSA solvent), or tranylcypromine and TSA (T+TSA). (E) Histone H3 visualized on a coomassie gel was used for normalization. Tranylcypromine alone does not affect global H3 acetylation (A,B) or H3K4 methylation (C,D) in GC-1 cells. TSA exposure causes a global increase in H3K9 (A) and total H3 acetylation (B) and, probably due to an indirect effect [91], H3K4 dimethylation (D). The combined treatment of T+TSA induces a global increase in H3K4 monomethylation (C), but does not further enhance the effect on H3 acetylation (A,B) or H3K4 dimethylation (C). Signals from proteins isolated from control cells served as the base and were defined as one. Fold change indicates the ratio of signal intensity in treated cells normalized to histone H3 over signal intensity in control cells normalized histone H3. Data are expressed as mean ± SEM; **p<0.01, ***p<0.001. Means with different letters are significantly different. N indicates the number of independent experiments.
(2.21 MB EPS)

Figure S2 Tranylcypromine and trichostatin A do not affect global histone H3K9 methylation or H3K4 trimethylation levels. Western blot analyses of global histone (A) H3K9 mono-, (B) H3K9 di-, (D) H3K9 tri-, and (E) H3K4 trimethylation on protein extracts isolated from GC-1 cells either grown in culture medium (control, ctr), or treated with tranylcypromine (T), trichostatin A (TSA), or dimethylsulphoxid (DMSO; TSA solvent), or tranylcypromine and TSA (T+TSA). (C) and (F) Histone H3 visualized on coomassie gels was used for normalization. Signals achieved with proteins isolated from control cells served as base and were defined as one. Fold change indicates the ratio of signal intensity in treated cells normalized to histone H3 over signal intensity in control cells normalized histone H3. Data are expressed as mean ± SEM. N indicates the number of independent experiments.
(3.02 MB EPS)
