# Supplementary material for: Epigenetic Mechanisms Regulate Stem Cell Expressed Genes Pou5f1 and Gfra1 in a Male Germ Cell Line
Source: PLoS One. 2010 Sep 14;5(9):e12727. doi: 10.1371/journal.pone.0012727 (PMC2939054; doi:10.1371/journal.pone.0012727)
Supplement: Table S1 — Antibodies used for Western blotting (WB) and chromatin-immunoprecipitation (ChIP). (0.04 MB DOC) [file pone.0012727.s001.doc]

**Table S1:**

Antibodies used for Western blotting (WB) and chromatin-immunoprecipitation (ChIP).

| **Antibody** | **Catalogue ID** | **Company** | **Concentration** | **Use** |
| --- | --- | --- | --- | --- |
| Rabbit polyclonal to histone H3K4me1 | ab8895 | Abcam | 1:500 | WB |
| Rabbit polyclonal to histone H3K4me2 | ab7766 | Abcam | 1:800 | WB |
| Rabbit polyclonal to histone H3K4me3 | ab8580 | Abcam | 1:800 | WB |
| Anti-monomethyl Histone H3 (Lys9) | 07-450 | Milliopore | 1:2000 | WB |
| Anti-dimethyl Histone H3 (Lys9) | 07-441 | Milliopore | 1:1000 | WB |
| Anti-trimethyl Histone H3 (Lys9) | 07-442 | Milliopore | 1:2000 | WB |
| Anti-acetyl-Histone H3 (Lys9) | 07-352 | Milliopore | 1:2000 | WB |
| Anti-acetyl-Histone H3 | 06-599 | Milliopore | 1:5000 | WB |
| Rabbit polyclonal to LSD1 | ab17721 | Abcam | 1:500 | WB |
| Anti-HDAC1, clone 2E10 | 05-614 | Milliopore | 1:1000 | WB |
| Monoclonal anti-beta-Actin, clone AC-15 | A1978 | Sigma-Aldrich | 1:10000 | WB |
| Donkey anti-rabbit IgG HRP-conjugated | 711-035-152 | Jackson IR Lab. | 1:5000 | WB |
| Donkey anti-mouse IgG HRP-conjugated | 715-035-150 | Jackson IR Lab. | 1:20000 | WB |
| Anti-H3K4me1 | ab8895 | Abcam | 5μg AB/50μg protein | ChIP |
| Di-Methyl-Histone H3 (Lys4) | 9726 (Lot 1) | Cell signaling | 5μl AB/50μg protein | ChIP |
| Anti-H3K9me1 | ab8896 | Abcam | 5μg AB/50μg protein | ChIP |
| Anti-acetyl-Histone H3 (Lys9) | 07-352 (Lot 07-352) | Milliopore | 5μl AB/50μg protein | ChIP |
| Anti-acetyl-Histone H3 | 06-599 | Milliopore | 7.5μg AB/50μg prot. | ChIP |
| Normal Rabbit Serum | 011-000-120 | Jackson IR Lab. | adjusted to AB conc. | ctr |
| Rabbit Gamma Globulin | 011-000-002 | Jackson IR Lab. | adjusted to AB conc. | ctr |
| Mouse Gamma Globulin | 015-000-002 | Jackson IR Lab. | adjusted to AB conc. | ctr |
