# Supplementary material for: Epigenetic Mechanisms Regulate Stem Cell Expressed Genes Pou5f1 and Gfra1 in a Male Germ Cell Line
Source: PLoS One. 2010 Sep 14;5(9):e12727. doi: 10.1371/journal.pone.0012727 (PMC2939054; doi:10.1371/journal.pone.0012727)
Supplement: Table S2 — Primers and TaqMan gene expression assays used for qPCR or ChIP-qPCR. (0.07 MB DOC) [file pone.0012727.s002.doc]

**Table S2:**

Primers and TaqMan gene expression assays used for qPCR or ChIP-qPCR.

| **Name** | **Sequence 5’-3’** | **Use** |
| --- | --- | --- |
| mmGapdh-ChIP-fw1 | GGTGCGTGCACATTTCAAAA | ChIP-qPCR |
| mmGapdh-ChIP-re1 | CCCGTAAAGCCGCGAGTAG | ChIP-qPCR |
| mmGfra1-ChIP-fw3 (C) | GCGAGCTGGTCGGTCAAA | ChIP-qPCR |
| mmGfra1-ChIP-re3 (C) | GACACCCGCTGTCCTTTAGG | ChIP-qPCR |
| mmGfra1-ChIP-fw5 (D) | GCTAGGCAAGTGCGTGTGAA | ChIP-qPCR |
| mmGfra1-ChIP-re5 (D) | CTCCGCCCCTACTCCTACCT | ChIP-qPCR |
| mmGfra1-ChIP-fw10 (G) | GCCTTGGTGTGAAAATATGGAAA | ChIP-qPCR |
| mmGfra1-ChIP-re10 (G) | TGGAACTACTGTGGGAGATGGA | ChIP-qPCR |
| MyoD-chip-fw1 | GCCGGTGTGCATTCCAA | ChIP-qPCR |
| MyoD-chip-re1 | TCAACCCAAGCCGTGAGAGT | ChIP-qPCR |
| mmOct4-ChIP-fw2 (B) | CCCTTTGAACCTGAAGTCAGATATTT | ChIP-qPCR |
| mmOct4-ChIP-re2 (B) | GCCTAGTTCCTGGGTGGAGAA | ChIP-qPCR |
| mmOct4-ChIP-fw3 (A) | TGGTTGCAAAGCCAGTCACTA | ChIP-qPCR |
| mmOct4-ChIP-re3 (A) | TGACTCACTGGCCAGGACAA | ChIP-qPCR |
| mmOct4-ChIP-fw4 (E) | GGCAAGAAACTGGATCAGATGAG | ChIP-qPCR |
| mmOct4-ChIP-re4 (E) | CCTGGTCCCGAGCTGTGA | ChIP-qPCR |
| mmOct4-ChIP-fw8 (F) | GGGAAGCAGGGTATCTCCATCT | ChIP-qPCR |
| mmOct4-ChIP-re8 (F) | CCCCAATCCCCTCACACA | ChIP-qPCR |
| mm-c-Kit-ex1-fw1 | TGCTCTGCGTCCTGTTGGT | qPCR |
| mm-c-Kit-ex2-re1 | TGGACTTGCAGATGGCTGAGA | qPCR |
| mmCrem-ex5-fw1 | TTTCCTCTGATGTGCCTGGTATT | qPCR |
| mmCrem-ex5-re1 | TGGTGTCCCTTCTTCCTCTGA | qPCR |
| mmCyct-ex3-fw1 | GATGCAAACAAGAACAAAGGTGTTA | qPCR |
| mmCyct-ex4-re1 | TCGGGTTCTCCAAATATTCCAT | qPCR |
| mmGapdh-fw1 | AACGACCCCTTCATTGACCTC | qPCR |
| mmGapdh-re1 | TTGAATTTGCCGTGAGTGGA | qPCR |
| mmGfra1-ex8-fw1 | GAAATCCAATGTATCGGGCAGTA | qPCR |
| mmGfra1-ex9-re1 | GCGAGACCATCCTTTCCGTAA | qPCR |
| mmKlf4-ex2-fw1 | GCGGGAAGGGAGAAGACACT | qPCR |
| mmKlf4-ex3-re1 | TCGCTTCATGTGAGAGAGTTCCT | qPCR |
| mmLdhc4-ex2-fw1 | ACTGAAGGGTTTGGCTGATGA | qPCR |
| mmLdhc4-ex3-re1 | TCTCCCCTCAGTTTGTTCGTATC | qPCR |
| mmLdhc-ex6_fw1 | CCCATATGGAGTGGTGTAAACGT | qPCR |
| mmLdhc-ex6_re1 | TCCTATTGCTGGGTTCAGTGACT | qPCR |
| MyoD-fw1 | GGCGACTCAGATGCATCCA | qPCR |
| MyoD-re1 | GCTGTAATCCATCATGCCATCA | qPCR |
| mmNgn3-ex1-fw1 | CGTCTTTACTGCCCGCTACAT | qPCR |
| mmNgn3-ex2-re1 | TCGTGGACTAAGGCAGAATGG | qPCR |
| mmOct4-ex2-fw1 | CCTGGGCGTTCTCTTTGGA | qPCR |
| mmOct4-ex3-re1 | AGGCCTCGAAGCGACAGAT | qPCR |
| mmSycp3-ex1-fw1 | GCCGCTGAGCAAACATCTAAA | qPCR |
| mmSycp3-ex2-re1 | CCCAGATTTCCCAGAATGCTT | qPCR |
| mmZbtb16-ex2-fw1 | CAGTGGAGCAGCACAGGAAA | qPCR |
| mmZbtb16-ex3-re1 | CGCAGAGTTCACACCCGTATG | qPCR |
| Mm00658129_gH (Pou5f1/Oct4) | TaqMan Assay (ABS, Foster City, CA, USA) | qPCR |
| Hs99999901_s1 (18S) | TaqMan Assay (ABS, Foster City, CA, USA) | qPCR |
