# Supplementary material for: Epigenetic Mechanisms Regulate Stem Cell Expressed Genes Pou5f1 and Gfra1 in a Male Germ Cell Line
Source: PLoS One. 2010 Sep 14;5(9):e12727. doi: 10.1371/journal.pone.0012727 (PMC2939054; doi:10.1371/journal.pone.0012727)
Supplement: Table S3 — qPCR analysis of male germ cell marker gene expression in untreated GC-1 cells. (0.04 MB DOC) [file pone.0012727.s003.doc]

**Table S3:**

**qPCR Analysis of male germ cell marker gene expression in untreated GC-1 cells.**

|  | ***Testis*** |  | ***GC-1 cells*** |  | |
| --- | --- | --- | --- | --- | --- |
| **Gene** | **target gene**  **[Ct ± sem]** | **calibrator**  **[Ct ± sem]** | **target gene**  **[Ct ± sem]** | **calibrator**  **[Ct ± sem]** | |
| ***Pou5f1*** | PND6 (ref. tissue) | | GC-1 | | |
|  | **30.42 ± 0.11** | 13.99 ± 0.09  (18S rRNA) | **36.05 ± 0.22** | 13.99 ± 0.08  (18S rRNA) | |
| ***Gfra1*** | PND6 (ref. tissue) | | GC-1 | | |
|  | **27.67 ± 0.07** | 20.26 ± 0.08  (*Gapdh*) | **29.77 ± 0.27** | | 17.70 ± 0.1  (*Gapdh*) |
| ***Zbtb16*** | PND8 (ref. tissue) | | GC-1 | | |
|  | **22.78 ± 0.02** | 20.11 ± 0.06  (*Gapdh*) | **37.39 ± 0.93** | | 17.40 ± 0.04  (*Gapdh*) |
| ***c-Kit*** | PND8 (ref. tissue) | | GC-1 | | |
|  | **26.65 ± 0.08** | 20.23 ± (5.0E-05)  (*Gapdh*) | **36.78 ± 0.65** | | 17.57 ± 0.05  (*Gapdh*) |
| ***Klf4*** | adult testis (ref. tissue) | | GC-1 | | |
|  | **22.09 ± 0.01** | 20.90 ± 0.05  (*Gapdh*) | **24.01 ± 0.06** | | 17.58 ± 0.06  (*Gapdh*) |
| ***Ldhc*** | adult testis (ref. tissue) | | GC-1 | | |
|  | **17.74 ± (4.9E-05)** | 20.57 ± 0.08  (*Gapdh*) | **34.11 ± 0.15** | | 17.40 ± 0.04  (*Gapdh*) |
| ***Crem*** | adult testis (ref. tissue) | | GC-1 | | |
|  | **19.83 ± (6.5E-04)** | 20.76 ± 0.03  (*Gapdh*) | **28.15 ± 0.04** | | 17.44 ± 0.05  (*Gapdh*) |
|  |  |  |  |  |  |

The Cycle threshold (Ct) defines the cycle number at which the amount of amplified target crosses a fixed threshold. The threshold defines a level of fluorescent signal set above the baseline but sufficiently low to be in the exponential growth region of the amplification curve. The lower a Ct value the more copies of a transcript are present in a specific sample.
