# Supplementary material for: Epigenetic Mechanisms Regulate Stem Cell Expressed Genes Pou5f1 and Gfra1 in a Male Germ Cell Line
Source: PLoS One. 2010 Sep 14;5(9):e12727. doi: 10.1371/journal.pone.0012727 (PMC2939054; doi:10.1371/journal.pone.0012727)
Supplement: Table S4 — qPCR analysis of MyoD gene expression in untreated and treated GC-1 cells. (0.03 MB DOC) [file pone.0012727.s004.doc]

**Table S4:**

**qPCR analysis of *MyoD* gene expression in untreated and treated GC-1 cells.**

|  | ***Skeletal muscle*** |  | ***GC-1 cells*** |  | |
| --- | --- | --- | --- | --- | --- |
| **Gene** | **target gene**  **[Ct ± sem]** | **calibrator**  **[Ct ± sem]** | **target gene**  **[Ct ± sem]** | **calibrator**  **[Ct ± sem]** | |
| ***MyoD*** | skeletal muscle (ref. tissue) | | GC-1 (DMEM, untreated ctr) | | |
|  | **29.15 ± 0.04** | 19.43 ± 0.02  (Gapdh) | **39.45 ± 0.26** | 17.89 ± 0.08  (Gapdh) | |
|  |  | | GC-1 + DMSO | | |
|  |  |  | **39.77 ± 0.22** | | 17.92 ± 0.08  (Gapdh) |
|  |  | | GC-1 + Tranylcypromine | | |
|  |  |  | **39.34 ± 0.67** | | 18.16 ± 0.02  (Gapdh) |
|  |  | | GC-1 + TSA | | |
|  |  |  | **39.88 ± 0.11** | | 18.24 ± 0.03  (Gapdh) |
|  |  | | GC-1 + T + TSA | | |
|  |  |  | **39.68 ± 0.35** | | 18.37 ± 0.15  (Gapdh) |

The Cycle threshold (Ct) defines the cycle number at which the amount of amplified target crosses a fixed threshold. The threshold defines a level of fluorescent signal set above the baseline but sufficiently low to be in the exponential growth region of the amplification curve. The lower a Ct value the more copies of a transcript are present in a specific sample.
